# This World Is Yours

**Published:** 2005-12-15

**Authors:** Lynne S. Wilcox

*This land is your land, this land is my land, from California to the New York Island . . .* (1). When Woody Guthrie wrote and sang these lyrics in 1940, the song was more than a paean to a beautiful country. It was a call to recognize the inherent dignity of the common man. The song's passion arose from Guthrie's experiences during the Great Depression, when he traveled with other migrants from the poverty of the Oklahoma Dust Bowl to potential employment and a new life in California (2). The catastrophic economic events occurring in the country, combined with a terrible drought and harsh winds, forced families to abandon their farms and travel hundreds of miles, looking for work and gambling on a new future. Steinbeck's novel *The Grapes of Wrath* (3) is the epic story of this period in history.

Songs and stories are essential but not sufficient for transforming desperation into dignity. Public health plays a critical role in the conversion. In the initial issue of *Preventing Chronic Disease (PCD),* we announced that a goal of this journal is to encourage a dialogue between researchers and practitioners (4). Such communication moves public health forward from investigating the principles for health promotion to implementing those principles in programs for all citizens, including disenfranchised populations. This first issue of our third year illustrates another aspect of that dialogue: evaluating health programs to assess the effectiveness of these principles and practices. We thank Qaiser Mukhtar and Leonard Jack of the Division of Diabetes Translation, National Center for Chronic Disease Prevention and Health Promotion, Centers for Disease Control and Prevention, for serving as guest editors for this issue.

**Figure 1:**
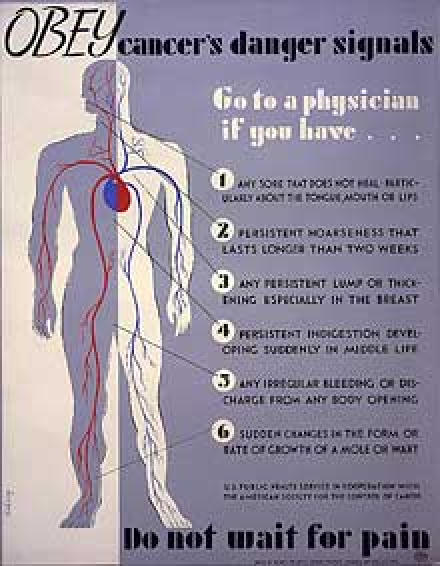
Cancer prevention poster from the Federal Art Project of the Works Progress Administration. (From Herzog H. Obey cancer's danger signals. 1938. Library of Congress,  Prints and Photographs Division. By the people, for the people: posters from the WPA, 1936-1943 [Internet]. Reproduction number cph 3g03639. Available from: URL: http://memory.loc.gov/ammem/ ndlpedu/collections/poster/file.html.)

**Figure 2:**
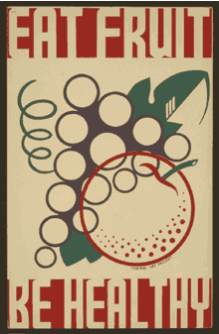
Healthy eating poster from the Federal Art Project of the Works Progress Administration. (From Eat fruit, be healthy. 1938. Library of Congress, Prints and Photographs Division. By the people, for the people: posters from the WPA, 1936-1943 [Internet]. Digital ID cph 3f05301. Available from: URL: http://memory.loc.gov/ammem/ ndlpedu/collections/poster/file.html.)

**Figure 3:**
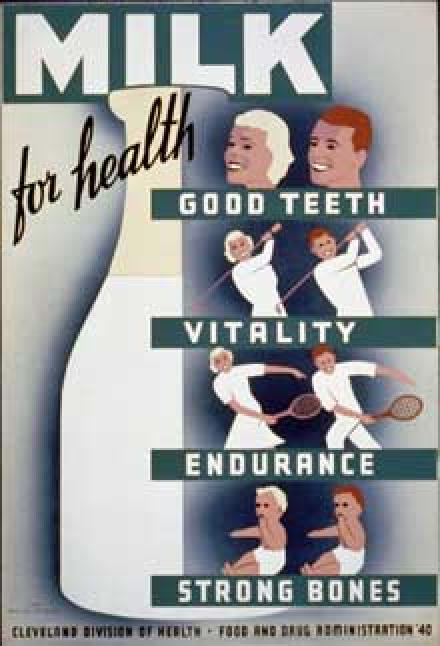
Milk promotion poster from the Federal Art Project of the Works Progress Administration. (From Milk for health. 1940. Library of Congress, Prints and Photographs Division. By the people, for the people: posters from the WPA, 1936-1943 [Internet]. Digital ID cph 3b48986. Available from: URL: http://memory.loc.gov/ammem/ ndlpedu/collections/poster/file.html.)

During the Great Depression, unemployment rates rose as high as 33%, and the Works Progress Administration (WPA) was formed to provide jobs and wages across the country. The WPA included the Federal Art Project, which hired artists to design posters for government programs such as public awareness campaigns supported by federal health agencies. Many of these posters addressed the prevention and control of chronic diseases. "Obey Cancer's Danger Signals" featured an outline of a human body and recommended consulting a physician for symptoms such as irregular bleeding, changes in the appearance of a mole, or lumps in the breast (Figure 1). The "Eat Fruit, Be Healthy" poster highlighted a drawing of grapes and an apple (Figure 2), and "Milk for Health" emphasized that milk consumption would result in good teeth and strong bones (Figure 3).

We know that these poster campaigns increased artists' incomes, but did they improve the health of U.S. citizens? World War II intervened before formal program evaluations could be conducted, although it remains unknown whether such evaluations were ever planned. Today, as Jack et al note, rigorous evaluations require recognition of real-world complexities and an approach that is multifaceted, multidisciplinary, and multidimensional (5).

Although we do not offer folk songs, over the past 2 years *PCD* has provided a forum for many voices. Our own evaluation indicates that we have published descriptions of public health programs from 37 states and research from 79 universities, federal or state agencies, and research centers. We are establishing an international presence: we now translate article abstracts into Spanish, French, and Chinese and have received Web site visitors from 67 countries.

Our hopes for *PCD* parallel Guthrie's aspirations for his music. "I am out to sing songs," Guthrie said, "that will prove to you that this is your world" (6).
